# Structural imbalance in youth weight management policies in China: a three-dimensional analysis of school responsibility and collaborative support

**DOI:** 10.3389/fpubh.2026.1941280

**Published:** 2026-08-25

**Authors:** Yun Zhang, Xiangyu Wang

**Affiliations:** Department of Physical Education, Capital Normal University, Beijing, China

**Keywords:** collaborative governance, health policy, policy instruments, school health promotion, weight management

## Abstract

**Objective:**

Youth weight management is a complex public health challenge requiring coordinated action across settings, with schools serving as primary implementation environments. This study examined how Chinese national policies allocate school responsibilities and configure external collaborative support.

**Methods:**

Thirty-six national policy documents issued in China between 2016 and 2025 were systematically analyzed. Unlike one-dimensional frequency analyses, the proposed framework linked policy targets, instruments, and implementing actors through three cross-dimensional co-occurrence matrices. NVivo-assisted coding, descriptive association indicators, and a sensitivity analysis were used to characterize policy-design patterns.

**Results:**

Environmental targets, capacity-building instruments, and schools were the most frequent categories. In the policy corpus, schools were overrepresented in pairings with command instruments and physical fitness targets, whereas external actors appeared in differentiated but weakly integrated pairings dominated by exhortation and capacity-building tools. Incentive and system-change instruments were comparatively uncommon. These textual patterns indicate that national policy design concentrates specified obligations in schools while providing relatively fewer structural and incentive mechanisms to connect schools with external actors.

**Conclusion:**

The three-dimensional framework identifies a school-centered policy design in which mandated tasks are more prominent than explicit mechanisms for systemic empowerment and cross-sector coordination. Policymakers can use the framework to compare assigned tasks with enabling instruments, clarify responsibility boundaries, establish school–health referral and coordination protocols, and match school mandates with earmarked resources. Because the analysis is limited to policy texts, the findings describe policy design rather than verified implementation or effectiveness.

## 1 Introduction

Childhood and adolescent weight management is a complex public health challenge that requires coordinated action across multiple ecological settings (1, 2). Schools provide a sustained institutional setting for youth health promotion, but school-based action depends on collaboration with families, health services, communities, and other sectors (3, 4). International reviews of whole-systems approaches and complex public health interventions similarly emphasize coordinated action across governance levels rather than isolated programs (5, 6). National policy frameworks shape this coordination by specifying goals, assigning responsibilities, and selecting instruments through which actors are expected to act. Analyzing how school responsibilities are configured alongside external support in policy texts is therefore important for understanding the design of youth weight-management governance.

International evidence positions schools as an important, but not self-sufficient, setting for preventing childhood and adolescent obesity. Policy, systems, and environmental interventions can improve school conditions, yet implementation varies substantially and commonly depends on trained personnel, leadership support, resources, and links to families and community or health services (7–11). These recurring challenges have been documented across different governance contexts, indicating that assigning tasks to schools is not equivalent to providing the conditions needed to carry them out. Policy content analysis offers a means of examining the institutional design that precedes implementation. Structured coding and text-mining studies classify policy themes, instruments, and actors to identify how responsibilities and resources are represented in formal documents (12, 13). Recent analyses of adolescent health and school-related policies have also used multidimensional frameworks to reveal imbalances that are obscured by aggregate frequency counts (14, 15).

Collaborative governance research conceptualizes cross-sector action as a set of institutional arrangements that connect public agencies and non-state stakeholders through shared purposes, roles, resources, and decision processes (16, 17). In China, recent policy studies have identified uneven policy-tool mixes and continuing needs to clarify stakeholder responsibilities in adolescent physical activity and community health governance (18, 19). However, most school-health policy analyses still report targets, instruments, or actors as separate frequency distributions. Treating schools as one actor among many does not show whether the targets assigned to schools are accompanied by enabling instruments or whether external actors are linked to complementary functions. A cross-dimensional approach is therefore needed to evaluate alignment within policy design. The present framework places school responsibility at the analytical center while retaining three distinct dimensions—policy targets, policy instruments, and implementing actors—and examines their pairwise co-occurrence patterns.

China provides a theoretically informative case because a centralized administrative system can support national coordination and rapid policy diffusion while also concentrating vertical responsibility within implementing organizations. This study examined the allocation of school responsibilities and the configuration of external collaborative support in 36 national policy documents on child and adolescent weight management issued between 2016 and 2025. It tested the following proposition: within the policy corpus, school actors would be overrepresented in co-occurrence with command instruments and physical fitness targets, whereas external actors would display segmented pairings and schools would receive comparatively limited incentive and system-change support. By linking targets, instruments, and actors across three matrices, the study moves beyond isolated frequency counts and evaluates whether assigned school responsibilities are textually aligned with enabling and collaborative mechanisms.

## 2 Theoretical framework

Policy design can be understood as the alignment of policy goals, governing instruments, and the actors expected to translate formal intentions into action. Policy instruments embody different assumptions about how behavior will be produced: authority-based tools prescribe action, incentives alter costs or benefits, capacity-building expands the ability to act, exhortation relies on information or persuasion, and system-change tools modify organizational or interorganizational arrangements (20, 21). A target-only analysis identifies what government seeks to change, while an instrument-only analysis identifies the selected means and an actor-only analysis identifies who is named. None of these dimensions alone shows whether an actor’s assigned targets are accompanied by instruments suited to those responsibilities. The three-dimensional framework therefore treats alignment—not frequency by itself—as the central theoretical property of policy design.

Youth weight management is particularly suited to this perspective because obesity emerges from interacting behavioral, institutional, commercial, and environmental conditions. Whole-systems and complex-systems research argues that isolated interventions cannot adequately represent these interdependencies and that coordinated action is needed across settings and levels of governance (5, 6). Collaborative governance theory further suggests that cross-boundary action depends on institutional arrangements that connect shared purpose, principled engagement, capacity for joint action, and mechanisms of accountability (16, 17). From this perspective, simply naming several actors in a policy does not demonstrate collaboration. A stronger collaborative design would connect actors to complementary functions and provide instruments that support information exchange, resource mobilization, joint decision-making, and continuity across organizational boundaries.

International research on school-based obesity prevention illustrates this distinction between assigning a role and enabling it. Reviews of policy, systems, and environmental interventions show that schools can support nutrition, physical activity, and healthy environments, but implementation is heterogeneous and often depends on leadership, trained personnel, infrastructure, family participation, and connections to health or community services (7–11). These studies also caution against treating schools as autonomous delivery systems for a problem shaped by forces beyond the education sector. The relevant theoretical question is therefore not whether schools appear frequently in policy documents, but how school targets are paired with governing instruments and how external actors are configured around those targets. Because the present study analyzes policy texts, the framework evaluates the architecture of intended coordination; it does not measure whether collaboration occurred or whether student outcomes changed.

China’s centralized administrative structure makes this alignment question especially informative. Central coordination can facilitate nationwide agenda setting, common standards, interministerial directives, and rapid diffusion of policy priorities. At the same time, vertical transmission may concentrate responsibilities in organizations such as schools if mandates are more explicit than the resources or cross-sector mechanisms that accompany them. Recent Chinese policy studies have reported imbalances in instrument mixes and insufficient specification of stakeholder responsibilities in adolescent physical activity and community health governance (18, 19). Building on that literature, the present model generates three linked diagnostics: the instrument–target matrix characterizes the means attached to policy goals; the instrument–actor matrix characterizes how governing tools are distributed among organizations; and the target–actor matrix characterizes substantive responsibility allocation. Their joint interpretation permits a more explicit assessment of whether school obligations are textually matched by enabling tools and collaborative support.

## 3 Methods

### 3.1 Data sources and policy selection

National policy documents related to child and adolescent weight management published between January 1, 2016, and December 31, 2025, were selected for analysis. Document retrieval was conducted through the official websites of the State Council of the People’s Republic of China, the National Health Commission, the Ministry of Education, and the General Administration of Sport of China. The inclusion criteria required that the documents were issued by national-level party and government departments or professional administrative institutions. The titles or main texts needed to address core topics including children and adolescents, weight, obesity, overweight, nutrition, physical activity, or health management. Eligible document types comprised formal texts such as planning outlines, action plans, guidelines, standards, notices, and laws or regulations. The content had to be directly relevant to weight management in children and adolescents. Duplicate publications, policy interpretations, news reports, and local-level documents were excluded. Ultimately, 36 policy documents were included.

### 3.2 Analysis procedures

#### 3.2.1 Coding framework and quality control

A three-dimensional coding framework comprising policy targets, policy instruments, and implementing actors was constructed from policy-design theory (20, 21). Policy targets were classified as individual-behavior, environmental-construction, or macro-level outcome targets and subdivided into eight tertiary categories: improving health literacy, enhancing physical fitness, promoting healthy lifestyles, strengthening management systems and services, creating supportive environments, reducing obesity prevalence, promoting health equity, and reducing socioeconomic burden. Instruments were classified as command, incentive, exhortation, capacity-building, or system-change tools. Actors comprised schools (including kindergartens), families, communities, medical and health institutions, government agencies, research and professional institutions, social and public-welfare organizations, media platforms, and relevant enterprises. Schools were used as the primary analytical filter within this framework rather than as a fourth coding dimension.

Following established document- and content-analysis procedures (22, 23), an independent sentence or clause expressing a complete policy intention was treated as the basic unit. Documents were organized by title, issuing authority, year, and source before coding. Operational definitions combined the actor named, the action specified, and the intended target. For example, a mandatory clause requiring schools to provide or ensure physical activity was coded as school × command instrument × enhancing physical fitness; a clause directing health institutions to provide screening or technical guidance was coded to the relevant health actor, target, and capacity-building function. When one sentence contained distinct actions for different actors or targets, it was divided into semantic segments and each segment was coded separately. Ambiguous wording was reviewed against category definitions and the clause’s operative action; multiple codes were retained only when the text expressed substantively distinct policy intentions.

Two researchers independently coded a random 20% sample of the policy documents. Software-based intercoder consistency analysis yielded a Cohen’s kappa of 0.76, indicating adequate agreement beyond chance (24). Coding discrepancies were discussed face to face, and the coding principles were confirmed before one researcher completed the remaining coding for the full policy corpus.

#### 3.2.2 Statistical analysis

Node coding, frequency statistics, and matrix coding queries were conducted in NVivo 11 Plus. Frequencies and proportions described the distributions of policy targets, instruments, and implementing actors. Three matrices were then constructed: policy instruments × policy targets, policy instruments × implementing actors, and policy targets × implementing actors. Chi-square statistics and Cramér’s *V* were reported as descriptive association indicators for categorical matrices (25), and adjusted standardized residuals (ASRs) identified cells with observed co-occurrence above or below the values expected from the matrix margins. Because one policy clause could receive multiple codes, co-occurrence reference points were not independent observations. Accordingly, *p* values are not interpreted as population-level inferential evidence; interpretation prioritizes the direction, magnitude, and consistency of textual co-occurrence patterns. A sensitivity analysis excluding policies published in 2024 assessed whether the main distributional patterns depended on that high-output year.

## 4 Results

### 4.1 Overall distribution characteristics

**Figure 1:**
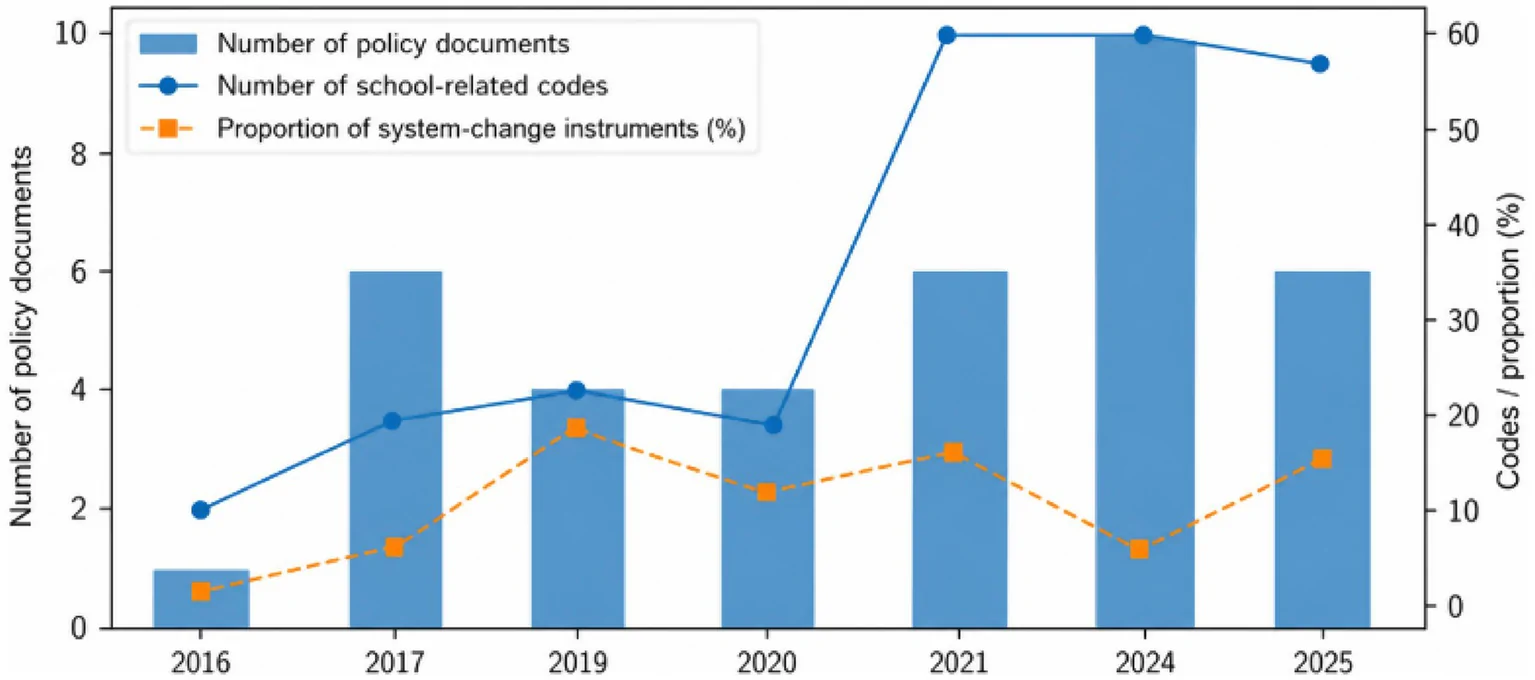
Trends in the number of included policy documents, school-related codes and the proportion of system-change instruments, 2016–2025.

Policy production peaked in 2024, with school-related coding also concentrated in the later study years (Figure 1). Across the full corpus, environmental targets were the most frequent target category, capacity-building and exhortation were the dominant instrument types, and schools were the most frequently coded implementing actor. These distributions establish a school-centered policy design, but frequency alone cannot determine whether school responsibilities are matched with particular instruments or external support; the cross-dimensional matrices address that question (Table 1).

**Table 1:** Distribution of policy targets, instruments and implementing actors in child and adolescent weight management policies.

Dimension	Category	Coding references	Percentage (%)
Policy targets	Individual behavioral targets	239	38.99
	Environmental targets	265	43.23
	Macro-level outcome targets	109	17.78
Policy instruments	Command instruments	145	16.37
	Incentive instruments	87	9.82
	Exhortation instruments	268	30.25
	Capacity-building instruments	274	30.93
	System-change instruments	112	12.64
Implementing actors	Schools (including preschools)	248	35.23
	Families (parents/caregivers)	97	13.78
	Communities (villages and neighborhood committees)	21	2.98
	Medical and health institutions	75	10.65
	Government agencies	141	20.03
	Research and professional institutions	37	5.26
	Social organizations and non-governmental organizations	27	3.84
	Media platforms	26	3.69
	Related enterprises	32	4.55

### 4.2 Structural associations among targets, instruments, and actors

The three matrices produced Cramér’s *V* values of 0.159–0.223, indicating modest descriptive clustering within the coded policy corpus. The instrument–target matrix emphasized exhortation for health literacy and healthy lifestyles, command tools for physical fitness, system-change tools for supportive environments, and capacity-building for management and services. The actor matrices showed differentiated policy roles: schools were overrepresented with command instruments and physical fitness targets; families and media with exhortation; medical and professional institutions with capacity-building; and communities, enterprises, and media with supportive-environment targets. These deviations identify design emphases in the documents and do not estimate implementation effects (Table 2).

**Table 2:** Descriptive association analysis of the three-dimensional coding matrices in child and adolescent weight management policies.

Analytical matrix	Co-occurrence references	*χ*^2^(df)	*P*-value	Cramér’s *V*	Major sources of difference (ASR > 1.96)
Policy instruments × Policy targets	751	149.71(28)	<0.001	0.223	Improving health literacy–Exhortation instruments (5.33); Enhancing physical fitness–Command instruments (5.30); Promoting healthy lifestyles–Exhortation instruments (5.18); Creating supportive environments–System-change instruments (4.08); Strengthening management systems and services–Capacity-building instruments (2.80)
Policy instruments × Implementing actors	1,022	131.91(32)	<0.001	0.180	Exhortation instruments–Families (parents/caregivers) (6.14); Exhortation instruments–Media platforms (5.54); Command instruments–Schools (including preschools) (4.06); System-change instruments–Social organizations and non-governmental organizations (3.27); Capacity-building instruments–Medical and health institutions (2.55)
Policy targets × Implementing actors	739	131.32(56)	<0.001	0.159	Enhancing physical fitness–Schools (including preschools) (4.73); Creating supportive environments–Media platforms (4.58); Reducing obesity prevalence–Medical and health institutions (3.93); Promoting healthy lifestyles–Families (parents/caregivers) (3.81); Creating supportive environments–Related enterprises (2.92)

Taken together, the model characterizes policy design through three linked questions: what goals are emphasized, which instruments are attached to those goals and actors, and how responsibility is distributed across organizations. Its contribution is not a new causal estimator but a reusable diagnostic structure. Future studies can apply the same matrices to other jurisdictions or time periods, compare national and local policy layers, or link text-based alignment scores with implementation evidence to test whether better-specified coordination is associated with practice.

### 4.3 Co-occurrence patterns for schools and external actors

For schools, capacity-building was the most frequent instrument in absolute terms, but command instruments were the only positively overrepresented tool pairing (ASR = 4.06), whereas system-change instruments were underrepresented (ASR = −2.78). Among school-related targets, creating supportive environments was most frequent, but enhancing physical fitness was the only positively overrepresented pairing (ASR = 4.73). A positive ASR therefore denotes a pairing that appears more often than expected from the matrix margins; it does not show that the associated policy was implemented or effective. Substantively, the school profile combines broad responsibilities with a disproportionate textual emphasis on directive fitness obligations (Table 3).

**Table 3:** Co-occurrence characteristics between schools and policy instruments/targets.

Analytical object	Category	Co-occurrence frequency	Percentage within group (%)	ASR
Schools × Policy instruments	Capacity-building instruments	113	35.2	−0.54
	Command instruments	74	23.1	4.06
	Exhortation instruments	69	21.5	−0.72
	Incentive instruments	36	11.2	0.04
	System-change instruments	29	9.0	−2.78
Schools × Policy targets	Creating supportive environments	67	25.2	−1.81
	Strengthening management systems and services	58	21.8	−0.77
	Promoting healthy lifestyles	46	17.3	1.13
	Enhancing physical fitness	36	13.5	4.73
	Improving health literacy	29	10.9	−0.13
	Reducing obesity prevalence	22	8.3	−0.84
	Promoting health equity	7	2.6	−0.41
	Reducing socioeconomic burden	1	0.4	−1.39

External actors showed differentiated pairings. Families and media were linked primarily to exhortation, medical and health institutions to capacity-building and obesity-prevalence targets, communities and enterprises to supportive environments, and social organizations to system-change instruments. These specialized pairings indicate how national documents distribute supporting functions, but the policy texts do not establish whether the roles were operationally connected in practice (Table 4).

**Table 4:** Co-occurrence characteristics of external collaborative actors with policy targets and instruments.

External actor	Policy instrument-related references	Policy target-related references	Primary associated policy target (ASR)	Primary associated policy instrument (ASR)
Families (parents/caregivers)	123	95	Promoting healthy lifestyles (3.81)	Exhortation instruments (6.14)
Medical and health institutions	112	78	Reducing obesity prevalence (3.93)	Capacity-building instruments (2.55)
Communities	40	22	Creating supportive environments (2.17)	Capacity-building instruments; Exhortation instruments
Social organizations and non-governmental organizations	53	26	Creating supportive environments	System-change instruments (3.27)
Related enterprises	48	23	Creating supportive environments (2.92)	Capacity-building instruments; Command instruments
Media platforms	41	28	Creating supportive environments (4.58)	Exhortation instruments (5.54)

### 4.4 Sensitivity analysis

After exclusion of the 2024 documents, environmental targets, capacity-building instruments, and schools remained the leading categories. Exhortation declined from 30.25 to 22.77%, indicating that advocacy-oriented language was more concentrated in 2024, while the main school-centered distribution was not dependent on that year (Table 5).

**Table 5:** Sensitivity analysis of key findings after excluding policy documents issued in 2024.

Dimension	Category	Full sample n (%)	Excluding 2024 documents n (%)	Interpretation
Policy targets	Environmental targets	265 (43.23)	211 (47.74)	Remained the most frequent policy target category
	Individual behavioral targets	239 (38.99)	158 (35.75)	Proportion decreased but remained the second most frequent category
Policy instruments	Capacity-building instruments	274 (30.93)	197 (31.37)	Remained the most frequently identified policy instrument
	Exhortation instruments	268 (30.25)	143 (22.77)	Proportion decreased, suggesting that 2024 policy documents contributed substantially to advocacy-oriented instruments
	System-change instruments	112 (12.64)	92 (14.65)	Proportion slightly increased after exclusion, while the interpretation regarding governance mechanism development remained unchanged
Implementing actors	Schools (including preschools)	248 (35.23)	188 (37.52)	Remained the most frequently coded implementing actor
	Government agencies	141 (20.03)	123 (24.55)	Remained an important coordinating actor

## 5 Discussion

This analysis identifies a school-centered architecture in China’s national youth weight-management policies. Schools were the most frequently coded actor and were positively overrepresented with command instruments and physical fitness targets. External actors were assigned differentiated functions, but the policy corpus contained relatively few incentive and system-change mechanisms connecting those functions. These findings support the proposed policy-design imbalance while remaining bounded to textual co-occurrence: they show how responsibilities and instruments are specified in national documents, not how policies were implemented or whether they improved health outcomes.

The school-centered pattern is consistent with the strategic importance of educational settings, but international research indicates that sustained school-based action usually depends on multicomponent programs, trained personnel, and organizational support rather than mandates alone (26, 27). The three-dimensional model adds to that literature by locating the potential mismatch within formal policy design. Absolute frequencies show that schools receive capacity-building provisions, whereas the residual profile shows that directive fitness obligations are disproportionately associated with schools and system-change tools are comparatively underrepresented. The appropriate interpretation is therefore an imbalance in the mix of specified tools, not proof of administrative overload experienced by schools.

A balanced interpretation must also recognize the potential strengths of centralized governance. National coordination can establish common standards, elevate youth health on the policy agenda, align ministries around shared priorities, and diffuse policy rapidly across jurisdictions. The concentration of policy issuance in recent years and the persistence of the main distributions after excluding 2024 are compatible with strong agenda-setting and standardization capacity. At the same time, accountability systems can become overly directive when responsibilities are specified more precisely than enabling arrangements, a tension also reported in research on physical-education accountability (28). Whether this tension affects implementation requires evidence from schools and local agencies.

The instrument profile further clarifies the design issue. Capacity-building and exhortation were prominent, whereas incentive and system-change instruments were less common. International reviews of obesity policy and complex public health systems emphasize that durable change often combines education and service capacity with fiscal, regulatory, environmental, and organizational mechanisms (6, 29). The current distribution therefore suggests an opportunity to diversify the policy mix. It does not demonstrate that existing exhortation or training was ineffective, nor does it establish why particular instruments were selected.

External-actor pairings indicate a potentially useful division of labor: families and media support lifestyle communication, health institutions contribute screening and technical capacity, and communities, enterprises, and social organizations contribute to supportive environments. However, collaborative governance requires more than differentiated roles; it also requires shared procedures, information flows, resources, and accountability across organizational boundaries (16, 17). International and Chinese studies of community initiatives, national fitness policy, and school–health partnerships similarly identify stable leadership, resourcing, and operational integration as important design elements (30–34). The limited textual specification of such linkages in the analyzed corpus is best interpreted as a policy-design gap; it should not be taken as evidence that informal or local collaboration is absent.

Several concrete mechanisms follow from this diagnosis. National guidance could assign a lead and co-lead agency for each school-related target, define a school–health referral protocol for screening and follow-up, and specify service-return and escalation responsibilities. Earmarked or pooled funds could support school coordinators, health-professional time, training, and community programs. Although derived from a different national and macroeconomic context, evidence from Pakistan suggests that health and education investment can complement labor-force participation in supporting economic growth, providing a broader rationale for coordinated investment across these sectors (35). A shared minimum monitoring dataset—with role-based access, privacy safeguards, and feedback to schools—could connect education and health information without treating schools as the sole data owner. Finally, incentive and system-change tools could be matched to high-burden mandates through protected staff time, cross-sector performance indicators, joint review meetings, and local adaptation authority.

The study’s contribution is an analytical framework for examining whether policy goals, instruments, and actor assignments are mutually aligned. Its limitations constrain interpretation. The corpus contains national Chinese policies and excludes local documents; coding reference points can overlap; instrument strength and resource intensity were not quantified; and policy texts cannot establish implementation, institutional performance, or outcomes. Future research should apply the model to national–local policy chains and comparative governance settings, test coding transferability, and combine document analysis with school case studies, administrative data, and interviews with education, health, family, and community stakeholders. Such work can evaluate whether textually aligned policy designs translate into coordinated practice.

## 6 Conclusion

This study examined how 36 Chinese national policies issued between 2016 and 2025 allocate school responsibilities and configure external support for child and adolescent weight management. Environmental targets, capacity-building instruments, and schools were the most frequent categories. Cross-dimensional matrices showed that schools were positively overrepresented with command instruments and physical fitness targets, whereas external actors appeared in specialized pairings and incentive and system-change support was comparatively limited.

The study advances policy analysis by treating targets, instruments, and actors as linked rather than isolated dimensions. The resulting model distinguishes overall policy attention from relational alignment and provides a reusable diagnostic for identifying where assigned responsibilities are or are not accompanied by enabling tools. Theoretically, it connects policy-instrument analysis with collaborative governance by operationalizing cross-sector policy design as observable actor–instrument–target configurations.

For policy development, the findings support more explicit responsibility and resource matching. Priority mechanisms include a formal school–health referral pathway, named lead and co-lead agencies, earmarked or pooled support for coordination, interoperable monitoring with privacy safeguards, and cross-sector indicators that reward joint action. These mechanisms would complement, rather than replace, the coordination and standard-setting advantages of centralized governance.

The main analytical constraints arose from multi-semantic policy clauses, overlapping codes, and the inability of national documents to reveal local practice. Semantic segmentation, operational definitions, duplicate coding, and sensitivity analysis strengthened internal consistency, but they cannot overcome the absence of implementation data. Accordingly, the reported associations characterize policy design and should not be interpreted as evidence of policy effectiveness or institutional performance.

Future studies can apply the three-dimensional model to local policy documents, other countries, or successive policy periods and assess its coding transferability. Linking the matrices with interviews, school-level cases, administrative indicators, and longitudinal outcomes would allow researchers to test whether stronger textual alignment among targets, instruments, and actors is associated with more coordinated implementation and more equitable youth weight-management support.

## Data Availability

The original contributions presented in the study are included in the article/Supplementary material, further inquiries can be directed to the corresponding author.
